# Analgesic Effects of Sevoflurane and Isoflurane on Elderly Patients with Colon Cancer and Their Influences on Immunity and Postoperative Cognitive Function

**Published:** 2019-03

**Authors:** Mingde YANG, Yan YU, Quanfeng LIU

**Affiliations:** 1. Department of Anesthesiology, Yidu Central Hospital of Weifang, Qingzhou 262500, P.R. China; 2. Department of Nursing, Yidu Central Hospital of Weifang, Qingzhou 262500, P.R. China

**Keywords:** Sevoflurane, Isoflurane, Elderly colon cancer, Anesthesia

## Abstract

**Background:**

We aimed to investigate the analgesic effect and immune function of elderly patients with colon cancer after application of sevoflurane and isoflurane anesthesia.

**Methods:**

Overall, 130 patients with colon cancer in Yidu Central Hospital of Weifang (Weifang, China) from February 2014 to January 2017 were collected and randomly divided into sevoflurane group (SEV group) and isoflurane group (ISO group). The pain score, immune indexes, postoperative cognitive index, extubation time, awakening time and S100R protein were analyzed.

**Results:**

The pain scores in SEV group at 5 min, 1 h and 3 h during surgery were significantly lower than those in ISO group (*P*=0.001, respectively). The levels of IL-6 in both groups of patients were higher at T1 and T2 than those at T0 (*P*=0.001). The levels of TNF-α in SEV group at T2 and T3 were significantly higher than that at T0 (*P*=0.001). The levels of CD80 in both groups of patients at T2 and T3 were obviously higher than those at T0 (*P*=0.001). Moreover, the extubation time, the response time to language and awakening time in SEV group were also remarkably shorter than those in ISO group (*P*=0.001). After continuous anesthesia in both groups of patients, the degrees of decline in ISO group were significantly higher than those in SEV group (*P*=0.001).

**Conclusion:**

Sevoflurane has a superior anesthetic effect to isoflurane in elderly patients with colon cancer, can reduce the degree of pain, improve the awakening condition and increase the immune function, so it is worthy of clinical application.

## Introduction

Colon cancer is a kind of tumor with a high morbidity rate in the elderly, which can be cured only by surgery (1–4). To reduce the pain of patients during surgery, anesthetics need to be applied, but the cognitive ability will be prone to decline after general anesthesia in clinic, and immune dysfunction and other adverse reactions will be caused (5–8). In order to avoid and reduce the occurrence of the above adverse reactions, therefore, it is particularly critical to choose anesthetics. At present, sevoflurane is often used for anesthesia in clinic. However, there is little related research on this anesthetic agent (9, 10).

In this study, 130 elderly patients with colon cancer in Yidu Central Hospital of Weifang from February 2014 to January 2017 were selected and anaesthetized with sevoflurane and isoflurane, respectively, so as to study the anesthetic effect of sevoflurane in elderly patients with colon cancer.

## Materials and Methods

### General data

A total of 130 elderly patients with colon cancer in Yidu Central Hospital of Weifang (Weifang, China) from February 2014 to January 2017 were randomly divided into SEV group (anesthesia via sevoflurane) and ISO group (anesthesia via isoflurane). There were 67 patients in SEV group, including 34 males and 33 females aged 65–79 yr old with an average of (70.32±5.42) yr old. There were 63 patients in ISO group, including 32 males and 31 females aged 64–80 yr old with an average of (71.02±5.92) yr old.

This study was approved by the Ethics Committee of Yidu Central Hospital of Weifang.

Exclusion criteria: patients with mental disease who were unable to cooperate in clinical trials, or patients with language communication disorder.

### Methods

At half an hour before surgery, all patients were intramuscularly injected with atropine at a dose of 0.3 mg; after which hemodynamic indexes of patients, such as heart rate (HR), systolic blood pressure (SBP), diastolic blood pressure (DBP) and saturation of pulse oxygen (SpO_2_), should be monitored based on the test protocol on a regular basis. Anesthesia should be terminated immediately if any abnormality was found. Midazolam (0.05 mg/kg), vecuronium (0.1 mg/kg), propofol (1.0 mg/kg) and remifentanil (2 μg/kg) were intravenously injected into patients for anesthesia induction, during which the anesthetic effect on patients should be observed at all times. Moreover, patients received mechanical ventilation via tracheal intubation, before which relevant parameters were set: Under normal circumstances, respiratory rate was set as 12 times/min, tidal volume of mechanical ventilation as about 8 mL/kg, end-tidal carbon dioxide partial pressure at a reasonable range (35–40 mmHg generally), and inspiration/expiration ratio as about 0.5. Anesthesia was maintained using sevoflurane in SEV group, and using isoflurane in ISO group. The minimum alveolar concentration (MAC) was set as 1–2. Maintenance of anesthesia was terminated after the surgery was completed.

### Observation indexes

Pain score: The pain degrees of patients at 5 min, 1 h, 3 h and 24 h after surgery were evaluated, respectively. Visual analogue scale (VAS) was used as the scoring method, in which patient’s pain was graded from 0 to 10 points.

Immune indexes: The expression levels of inter-leukin-6 (IL-6), tumor necrosis factor-α (TNF-α) and soluble IL-2R (sIL-2R), and cell surface markers, cluster of differentiation 80 (CD80) and CD86, in patients were detected before anesthesia (T0), at 1 h during surgery (T1), at the end of surgery (T2), at 24 h after surgery (T3) and at 72 h after surgery (T4). The levels of serum IL-6, TNF-α and sIL-2R were detected via enzyme-linked immunosorbent assay (ELISA), and expression levels of cell surface markers, CD80 and CD86, in serum were detected by using a flow cytometer.

Cognitive function indexes: Mini-mental state examination (MMSE) was adopted for the evaluation of cognitive function of patients, mainly including 30 items, such as orientation, immediate short-term memory, attention, calculation, reading ability and verbal comprehension, and the total score is 30 points. The cognitive function of patients was evaluated by using MMSE before anesthesia and at 4 h, 24 h and 48 h after anesthesia, which should be finished within 8 min. The level of S100B protein in peripheral blood of patients was detected by using the ELISA kit.

### Data processing and analysis

Data obtained in this study were processed and analyzed by using Statistical Product and Service Solutions (SPSS) 17.0 software (Chicago, IL, USA). Measurement data were presented as (χ̄±s), and *t* test was used for the intergroup comparison of differences. Enumeration data were presented as [n (%)], and chi-square test was performed for the intergroup comparison of differences. *P*<0.05 suggested that the difference between two groups was statistically significant.

## Results

### Comparisons of intraoperative hemodynamic indexes (HR, SBP, DBP and SpO2) between the two groups of patients

HR, SBP, DBP and SpO_2_ values of patients in both groups at different time points (before surgery, after anesthesia, 1 h during surgery, 2 h during surgery and the end of surgery) were recorded, respectively. Results revealed that there were no significant differences in relevant indexes between the two groups of patients at each time point (Table 1).

**Table 1:** Comparisons of intraoperative hemodynamic indexes (HR, SBP, DBP and SpO_2_) between the two groups of patients (χ̄±s)

**Group**	**Index**	**Before surgery**	**After anesthesia**	**1 h during surgery**	**2 h during surgery**	**At the end of surgery**
SEV	HR (time/min)	82±3	86±7	80±5	85±7	83±5
	SBP (mmHg)	118±6	106±5	93±6	92±6	100±8
	DBP (mmHg)	86±5	67±3	65±5	66±5	87±6
	SpO_2_ (%)	99±7	99±2	95±7	95±6	95±9
ISO	HR (time/min)	83±4^*^	89±6^*^	80±6^*^	81±5^*^	84±7^*^
	SBP (mmHg)	117±7^*^	108±5^*^	91±5^*^	90±7^*^	105±8^*^
	DBP (mmHg)	86±6^*^	67±5^*^	66±3^*^	66±6^*^	88±6^*^
	SpO_2_ (%)	97±6^*^	100±7^*^	92±8^*^	94±7^*^	95±8^*^

* *P*>0.05 vs. SEV group

### Comparisons of pain scores between the two groups of patients

At 5 min, 1 h, 3 h and 24 h after anesthesia, pain scores were (2.3±0.7) points, (3.4±0.9) points, (5.2±0.9) points and (2.1±0.5) points, respectively, in SEV group, and they were (6.7±1.2) points, (7.9±1.3) points, (6.9±1.3) points and (2.2±0.9) points, respectively, in ISO group. The scores in SEV group at 5 min, 1 h and 3 h were significantly lower than those in ISO group (*P*=0.001) (Table 2).

**Table 2:** Comparisons of pain scores after anesthesia between the two groups of patients (χ̄±s, points)

***Group***	***n***	***5 min***	***1 h***	***3 h***	***24 h***
SEV	67	2.3±0.7	3.4±0.9	5.2±0.9	2.1±0.5
ISO	63	6.7±1.2^*^	7.9±1.3^*^	6.9±1.3^*^	2.2±0.9

* *P*<0.05 vs. SEV group

### Comparisons of IL-6, TNF-α and sIL-2R levels before and after surgery between the two groups of patients

The levels of IL-6 in both groups of patients were higher at T1 and T2 than those at T0, and the degrees of change in SEV group at T1 and T2 were significantly higher than those in ISO group (*P*=0.001). The levels of TNF-α in SEV group at T2 and T3 were significantly higher than that at T0, while there was no significant difference in the TNF-α level in ISO group at T2 compared with that at T0, but it was higher at T3 than that at T0 (*P*=0.001). The level of sIL-2R had no significant difference between the two groups of patients, and no significant difference was found at each time point (Table 3).

**Table 3:** Comparisons of IL-6, TNF-α and sIL-2R levels before and after surgery between the two groups of patients (χ̄±s)

***Group***	***Index***	***T0***	***T1***	***T2***	***T3***	***T4***
SEV	IL-6 (ng/L)	73.1±4.4	143.2±12.3#	103.2±9.4#	73.1±7.5	68.9±4.2
	TNF-α (ng/L)	43±5.2	41±3.4	69±3.6	67±8.9	47±2.9
	sIL-2R (U/mk)	552±12.3	549±13.3	563±12.3	572±10.5	578±10.5
ISO	IL-6 (ng/L)	71.9±4.2	139.2±10.3#	159.2±10.8^*^#	81.3±4.2	69.3±3.2
	TNF-α (ng/L)	42±2.3	39±2.4	44±2.3^*^	77±5.8	45±2.7
	sIL-2R (U/mk)	559±15.4	568±10.9	572±11.9	569±10.3	574±10.7

# *P*<0.05 vs. at T0

* *P*<0.05 vs. SEV group

### Comparisons of CD80 and CD86 levels before and after surgery between the two groups of patients

The levels of CD80 in both groups of patients at T2 and T3 were higher than those at T0. The difference in the level of CD80 compared with that before anesthesia in SEV group was obviously smaller than that in ISO group (*P*=0.001). There were no significant differences in changes in the CD86 level between the two groups of patients at different time points (Table 4).

**Table 4:** Comparisons of CD80 and CD86 levels before and after surgery between the two groups of patients

***Group***	***CD80***			***CD86***		
	***Before anesthesia***	***At the end of surgery***	***24 h after surgery***	***Before anesthesia***	***At the end of surgery***	***24 h after surgery***
SEV	3.52±1.21	1.79±0.21^*^	1.76±0.09^*^	18.21±1.42	16.38±1.13	17.31±1.23
ISO	3.42±1.09	1.53±0.19^*^#	1.52±0.05^*^#	16.52±1.08	15.78±0.92	16.05±1.02

# *P*<0.05 vs. before anesthesia //

* *P*<0.05 vs. SEV group

### Comparison of awakening condition between the two groups of patients

It was found after anesthesia that respiratory recovery time, recovery time of pharyngeal reflex and operation time had no significant differences between the two groups of patients (*P*=1.421,0.975,0.653, respectively). The extubation time in SEV group was obviously shorter than that in ISO group, and the response time to language and awakening time in SEV group were also remarkably shorter than those in ISO group, with statistically significant differences (*P*=0.001) (Table 5).

**Table 5:** Comparison of awakening condition between the two groups of patients (χ̄±s, min)

***Group***	***n***	***Respiratory recovery time***	***Recovery time of pharyngeal reflex***	***Extubation time***	***Operation time***	***Response time to language***	***Awakening time***
SEV	67	4.3±2.8	5.2±0.93	7.9±1.07	112±10.24	6.93±2.95	6.21±2.04
ISO	63	4.3±1.6	5.5±2.16	20.21±3.46^*^	114±10.83	15.21±1.95^*^	14.56±1.64^*^

* *P*<0.05 vs. SEV group

### Comparisons of cognitive levels before and after anesthesia between the two groups of patients

MMSE scores at 4 h and 24 h after anesthesia in both groups of patients were remarkably decreased, with statistically significant differences (*P*=0.001), and the degrees of decline in ISO group were significantly higher than those in SEV group (*P*=0.001) (Table 6).

**Table 6:** Comparisons of MMSE scores before and after anesthesia between the two groups of patients (χ̄±s, min)

***Group***	***n***	***Before anesthesia***	***4 h after anesthesia***	***24 h after anesthesia***	***48 h after anesthesia***
SEV	67	28.92±1.86	27.23±1.98^*^	26.98±2.01^*^	28.98±2.01
ISO	63	29.01±2.43	23.21±1.55^*^#	24.05±1.33^*^#	29.01±2.31

* *P*<0.05 vs. before anesthesia //

# *P*<0.05 vs. SEV group

### Comparisons of S100B protein levels before and after anesthesia between the two groups of patients

The levels of S100B protein at 4 h and 24 h after anesthesia in both groups of patients were remarkably higher than those before anesthesia, and the degrees of increase in ISO group were significantly higher than those in SEV group, with statistically significant differences (*P*=0.001) (Table 7).

**Table 7:** Comparisons of S100B protein levels before and after anesthesia between the two groups of patients (χ̄±s, ng/mL)

***Group***	***n***	***Before anesthesia***	***4 h after anesthesia***	***24 h after anesthesia***	***48 h after anesthesia***
SEV	67	1.01±0.09	1.18±0.11^*^	1.27±0.88^*^	1.01±0.55
ISO	63	0.99±0.09	1.37±0.09^*^#	1.46±0.10^*^#	1.02±0.54

* *P*<0.05 vs. before anesthesia //

# *P*<0.05 vs. SEV group

## Discussion

The incidence rate of elderly colon cancer is high, and patients should undergo surgery as soon as possible once diagnosed. To reduce the pain of patients during surgery and to ensure the smooth surgery, general anesthesia is needed for patients (11–13). However, it is clinically believed that anesthesia can cause damage to the patient’s immune system. Relevant studies have demonstrated that after patients are anesthetized, the anesthetics can inhibit the T cells in the body, thus affecting the immune function. IL-6 and TNF-α are the main factors in the diagnosis of immune function. IL-6 is involved in the nerve-endocrine-immune system of human body, and TNF-α can promote T lymphocytes to exert the immune function, both of which, therefore, are important indexes in evaluating the patient’s immune response function (13–16). Besides, CD80 and CD86 in the body can enhance the immune response of cells in vivo to tumor cells. When these two antibodies are decreased in vivo, the response function of T cells will be weakened, which will affect the body’s immune function.

Anesthesia in elderly patients will affect the patient’s central nervous system, resulting in cognitive impairment. MMSE is generally applied in the evaluation of cognitive function, which is more accurate because it can rule out such factors as unconsciousness and emotional fluctuation of patients (17, 18). In addition, the index S100B protein, a kind of neurotrophin secreted by the central nervous system, is also used, which plays a key role in the cognitive function of the human body. It is clinically found that when the nervous system is damaged, S100B protein will enter the cerebrospinal fluid through the damaged barrier, leading to an elevated level of protein in the serum. Moreover, the increase in S100B protein indicates severe damage to the nervous system.

Nowadays, isoflurane is one of the clinically-used anesthetics, which is mainly applied in semi-general anesthesia and general anesthesia, and sufficient oxygen inhalation can be provided during anesthesia. However, the pain degree of patients is less reduced after isoflurane is used, and there is also a certain effect on cognitive function. Besides, sevoflurane is widely used in clinic now, and it can be absorbed quickly. Patients can wake up earlier, and the patient’s respiratory tract will be less damaged after inhalation of sevoflurane, so it is suitable for patients with cardiovascular disease. In addition to its anesthetic effect, sevoflurane will also inhibit the synaptic transmission of cholinergic neurons, thereby reducing the damage to cognitive function of patients (19, 20).

In this study, the pain score of patients receiving sevoflurane during surgery was significantly lower than that of patients receiving isoflurane, suggesting that sevoflurane anesthesia has a better anal-gesic effect, and can alleviate the pain of patients during surgery. In addition, the levels of immune indexes (IL-6 and TNF-α) in patients receiving sevoflurane were higher than those in patients receiving isoflurane at several time points, indicating that sevoflurane can enhance the immune response of T cells, thus strengthening the immune function. MMSE scores of all patients at 4 h and 24 h after anesthesia were decreased compared with those before anesthesia, showing statistically significant differences (*P*=0.001), and the degrees of decline in ISO group were remarkably higher than those in SEV group (*P*=0.001), indicating that both sevoflurane and isoflurane will affect the patient’s cognitive function, the former of which has a smaller impact on cognitive ability than isoflurane, thus avoiding huge damage to cognitive ability. Furthermore, the awakening time and extubation time of patients receiving sevoflurane were obviously shorter than those of patients receiving isoflurane (*P*=0.001), suggesting that the recovery is faster after sevoflurane anesthesia with a shorter impact on cognitive function. Ma Zhijia et al compared the anesthetic effect of sevoflurane and propofol on patients undergoing laparoscopic cholecystectomy and found that the awakening time and pain score of patients receiving sevoflurane were significantly shorter and lower than those of patients receiving propofol, and the consciousness recovery of patients after maintenance of anesthesia using sevoflurane was faster, and the analgesic effect of sevoflurane was more obvious, so it is more suitable for anesthesia in such operations as laparoscopic cholecystectomy (21). Bu Yebo et al found through experiments that the cognitive function score of elderly patients receiving sevoflurane during surgery was significantly higher than that of patients receiving isoflurane (22).

## Conclusion

Sevoflurane has a better clinical effect in maintenance of anesthesia for elderly patients with colon cancer, can reduce the pain of patients, avoid the damage to the body immunity, and reduce the impact on cognitive function of patients, so it is suitable for anesthetic treatment in surgery, and worthy of clinical promotion and application.

## Ethical considerations

Ethical issues (Including plagiarism, informed consent, misconduct, data fabrication and/or falsification, double publication and/or submission, redundancy, etc.) have been completely observed by the authors.
